# Antimicrobial Perspectives of Active SiO_2_Fe_x_O_y_/ZnO Composites

**DOI:** 10.3390/pharmaceutics14102063

**Published:** 2022-09-27

**Authors:** Florin Matusoiu, Adina Negrea, Nicoleta Sorina Nemes, Catalin Ianasi, Mihaela Ciopec, Petru Negrea, Narcis Duteanu, Paula Ianasi, Daniel Duda-Seiman, Delia Muntean

**Affiliations:** 1 Faculty of Industrial Chemistry and Environmental Engineering, Politehnica University Timisoara, Victoriei Square, No. 2, 300006 Timişoara, Romania or; 2 Renewable Energy Research Institute-ICER, Politehnica University Timisoara, 138 Gavril Musicescu Street, 300501 Timişoara, Romania; 3 “Coriolan Drăgulescu” Institute of Chemistry, Bv. Mihai Viteazul, No. 24, 300223 Timişoara, Romania; 4 National Institute for Research and Development in Electrochemistry and Condensed Matter, 144th Dr. A. P. Podeanu Street, 300569 Timişoara, Romania; 5 Department of Cardiology, “Victor Babeş” University of Medicine and Pharmacy Timişoara, 2 Piata Eftimie Murgu, 300041 Timişoara, Romania; 6 Multidisciplinary Research Centre on Antimicrobial Resistance, Department of Microbiology, “Victor Babeş” University of Medicine and Pharmacy Timişoara, 2 Eftimie Murgu Square, 300041 Timişoara, Romania

**Keywords:** antimicrobial activity, zinc oxide, composites, silica matrix

## Abstract

The antibacterial activity of zinc oxide particles has received significant interest worldwide, especially through the implementation of technology to synthesize particles in the nanometer range. This study aimed to determine the antimicrobial efficacy of silica-based iron oxide matrix (SiO_2_Fe_x_O_y_) synthesized with various amounts of ZnO (SiO_2_Fe_x_O_y_ZnO) against various pathogens. It is observed that, with the addition of ZnO to the system, the average size of the porosity of the material increases, showing increasingly effective antibacterial properties. Zinc-iron-silica oxide matrix composites were synthesized using the sol–gel method. The synthesized materials were investigated physicochemically to highlight their structural properties, through scanning electron microscopy (SEM), Energy Dispersive X-ray Spectroscopy (EDX), and Fourier-transform infrared spectroscopy (FT-IR). At the same time, surface area, pore size and total pore volume were determined for materials synthesized using the Brunauer–Emmett–Teller (BET) method. Although the material with 0.0001 g ZnO (600 m^2^/g) has the highest specific surface area, the best antimicrobial activity was obtained for the material with 1.0 g ZnO, when the average pore volume is the largest (~8 nm) for a specific surface of 306 m^2^/g. This indicates that the main role in the antibacterial effect has reactive oxygen species (ROS) generated by the ZnO that are located in the pores of the composite materials. The point of zero charge (pH_pZc_) is a very important parameter for the characterization of materials that indicate the acid-base behaviour. The pH_pZc_ value varies between 4.9 and 6.3 and is influenced by the amount of ZnO with which the iron-silica oxide matrix is doped. From the antimicrobial studies carried out, it was found that for *S. aureus* the total antibacterial effect was obtained at the amount of 1.0 g ZnO. For Gram-negative bacteria, a total antibacterial effect was observed in *S. flexneri* (for the material with 0.1 g ZnO), followed by *E. coli* (for 1.0 g ZnO). For *P. aeruginosa*, the maximum inhibition rate obtained for the material with 1.0 g ZnO was approximately 49%.

## 1. Introduction

Multifunctional composite materials have become an important research topic in recent years. These composite materials can be ideal for certain areas depending on their use. By combining crystalline, amorphous and polymeric phases, we can enrich and improve the properties of any final material [1].

Composite materials consisting of simple or mixed small metal oxide particles embedded in amorphous matrices, showing more interesting properties, especially in terms of magnetic, electrical and catalytic properties [2]. 

The properties of composites consisting of oxide particles dispersed in an inert matrix depend significantly on the type of metal oxide, the size and dimensional distribution of the particles, and also the morphology and porosity of the matrix.

Regarding metal oxides, various oxides (CuO, TiO_2_, Fe_x_O_y_, ZnO) have been studied. Of these, ZnO is of particular importance due to its low cytotoxicity, especially in human cells [3], due to its good selectivity [3], but also its stability over time [4], in contrast to iron oxides which are used due to their biocompatibility, but also for the magnetic properties they exhibit [5].

The sol–gel synthesis used is a successful technique used for composites preparation due to the advanced control over the composition, purity, homogeneity, size and properties of the dispersed particles [6]. Generally, the sol–gel technique is based on the hydrolysis and condensation of silicon alkoxides, the most widely used being tetraethylorthosilicate (TEOS), but recently many of their derivatives have been used. The oxide particles that want to be embedded in the silica matrix are either dispersed as such in the initial sols, or generated during the heat treatment of the composite by the thermal decomposition of some precursors initially introduced into the TEOS-H_2_O-C_2_H_5_OH system. There is a possibility that, due to the large surface area of the interface between the dispersed particles and the silica matrix during heat treatment, the interaction between the two components takes place, leading to the formation, at high temperature, but in small quantities, of the corresponding secondary phase M_2_SiO_4_ [7], where M can be replaced with any metal.

The introduction of iron oxides in the silica matrix can improve the properties of the newly obtained material, especially under acidic conditions, because hydroxyl groups can functionalize the iron oxide to form bonds with various biological ligands [8]. Iron oxide can take several polymorphic forms such as α-Fe_2_O_3_, γ-Fe_2_O_3_ and Fe_3_O_4_, also found in nature. Recently, Fe_3_O_4_-based nanomaterials have been used in many fields due to their magnetic properties, low toxicity, and biocompatibility [9]. 

There are a number of applications in which nanocomposites based on iron and silica oxides can be used with great success, namely: in the remediation of the polluted environment by acting as adsorbents [10,11], or catalyzers [12,13], sensors [14] and especially in medical field [15,16].

In order to improve the properties of the materials, we introduced ZnO into the reaction mass used for composite preparation. The addition of zinc oxide particles can influence the porosity of the materials while also improving the adsorption of the elements. Based on data from literature, it was observed that the addition of ZnO to the composition of materials based on iron oxides causes the formation of compounds that can be used in the photodegradation of organic dyes and other pollutants [17,18,19]. Similar compounds obtained from zinc oxide began to be used for the adsorption of heavy metals, for the obtaining of solar cells, gas sensors [20,21], for applications aimed at reducing microbiological contamination of wastewater, in terms of complete degradation of organic matter or chlorine, but also the control of the spread of infections with different microbial strains, combating multidrug resistance [22,23,24]. These successful applications are based on the properties of ZnO, such as high specific surface area, excellent hydrophobicity and good oxidation capacity. At the same time, ZnO in contact with microorganisms inhibits effectively their growth in the wastewater treatment and purification process [3]. The antimicrobial activity of ZnO can be optimized by using nanometer-sized ZnO particles, which ensure a large contact surface and improve interactions with microbial cells [4,25]. 

Anna Król-Górniak et al. explain how ZnO-doped materials exhibits much lower cytotoxicity in in vitro studies [26]. Other studies show that, although zinc-based materials can present beneficial properties, under certain conditions it must be taken into account that zinc is a risk element for the body. Once in the body, it can affect certain organs or tissues, and that is why the use of a magnetic material, based on Zn, can help by transporting the drugs directly to the target. Therefore, the goal of this study is the inclusion in the matrix of silica-iron oxides of different amounts of ZnO to improve the microbiological activity of the materials, in order to obtain active materials under antimicrobial aspect. 

## 2. Materials and Methods

### 2.1. Materials Synthesis and Characterization

To obtain composites based on the silica matrix and iron–zinc oxide, the synthesis was carried out by the sol–gel method in 3 stages: (i) in the first stage, 7 mL of tetraethyl-orthosilicate (TEOS), Si(OC_2_H_5_)_4_, (Sigma Aldrich, Saint Louis, MO, USA) were used as a silica precursor, together with 37.2 mL of ethanol used as a solvent (SC Chimopar Trading SRL, București, Romania), which were stirred for 10 minutes at 500 rpm, then 13.8 mL of distilled water were added, continuing the stirring for another 30 minutes; (ii) in the second step, 0.54 g of Fe(II) acetylacetonate, C_10_H_14_FeO_4_, Fe(acac)_2_ (SigmaAldrich, Saint Louis, MO, USA), which is the precursor for iron(II), together with ZnO powder (Merck, Darmstadt, Germany) in different amounts (0.0001 g ZnO, 0.001 g ZnO, 0.01 g ZnO, 0.1 g ZnO and 1.0 g ZnO), were added over the first solution and the obtained mixture was stirred for 3 h; (iii) in the third stage, hydrolysis and condensation of the material is possible by adding a catalyst, in our case 0.46 g of NH_3_ (SC Chimopar Trading SRL, Romania) was added, obtaining a solution, which before gelation has a PH = 10. To obtain the xerogel, the obtained material was dried for 24 h in an oven at 100 °C.

Obtained materials were characterized by scanning electron microscopy (SEM) coupled with energy dispersive X-ray spectroscopy (EDX), using the FEI Quanta FEG 250 X-ray energy dispersive spectrometer. Furthermore, the material was characterized by Fourier transform infrared spectroscopy (FT-IR) using Cary 630 Fourier transform infrared spectrophotometer KBr pellets. N_2_ adsorption–desorption isotherms are obtained using the Nova 1200e Quantachrome apparatus. Further, in order to confirm the crystallinity degree of the prepared material, the XRD spectra were recorded by using a Rigaku Ultima IV XRD diffractometer (Rigaku, Tokyo, Japan).

The zero load point, pH_pZc_, was also determined by the method of bringing the studied system to equilibrium. An amount of 0.1 g of material was used for this study, which was mixed with 25 mL of 0.1 N KCl solution (Merck, Darmstadt, Germany) at 200 rpm and a temperature of 298 K, using a water bath with a thermostat and stirring, a Julabo SW23 type. The pH of the KCl solutions was adjusted in the range of 1–14, using NaOH solutions (Merck, Darmstadt, Germany) with a concentration between 0.05 N and 2N or HNO_3_ solutions (Merck, Darmstadt, Germany) with a concentration between 0.05 N and 2 N. The samples were filtered and, subsequently, the pH of the resulting solution was determined using a pH meter of the METTLER TOLEDO, SevenCompact, S 210 type.

### 2.2. Microbiological Assays

Preparation of bacterial cultures for testing the antimicrobial effect of synthesized materials on a heterotrophic bacterial inoculum

To test the effectiveness of ZnO-based composite materials in terms of interaction with real microorganisms, we performed microbiological tests on a heterotrophic bacterial consortium, taken from the water of the Bega River. Inoculations were made in non-selective nutrient medium (Tryptone yeast extract agar ISO, VWR Chemicals for Microbiology, Leuven, Belgium), final pH 7.2 ± 0.2. Each set of experiments was performed with 3 replicates. The bacterial inoculum was adjusted to have the appropriate density for 0.5 McFarland (10^8^ CFU/mL). Then, 1 mL of the bacterial inoculum was taken and incorporated into the nutrient medium. Microbial cultures were carried out on materials without ZnO and with different ZnO content: 0.0001 g ZnO; 0.001 g ZnO; 0.01 g ZnO; 0.1 g ZnO; 1.0 g ZnO. Each time, the effect of 0.1 g of solid material was tested. After inoculation, plates were incubated for 48 h at 37 °C. Subsequently, the number of microbial colonies grown on each plate was determined using the automatic colony counter (Flash & Go, Yul Instruments, Barcelona, Spain).

### 2.3. Preparation of Bacterial Cultures for Testing the Antimicrobial Effect of Synthesized Materials on Reference Strains

To test the antimicrobial effect of the synthesized materials on the reference strains, microbiological control tests were performed using six reference strains from the American Type Culture Collection: *Staphylococus aureus* ATCC 25923 (Microbiologics, St. Cloud, MN, USA), *Candida parapsilosis* ATCC 22019 (Microbiologics, St. Cloud, MN, USA), *Pseudomonas aeruginosa* ATCC 27853 (Microbiologics, USA), *Escherichia coli* ATCC 25922 (Microbiologics, USA), *Shigella flexneri* ATCC 12022 (Microbiologics, St. Cloud, MN, USA). The bacterial inoculum was adjusted to have an optical density for 0.5 McFarland (10^8^ CFU/mL). From each microbial strain, 10 µL were taken (10^6^ CFU/mL) and the inoculum was deposited on the Petri dish containing test material. A control plate containing only the bacterial inoculum (10^6^ CFU/mL) was inoculated for each of the reference microbial strains. The bacterial cultures were incubated at 37 °C for 24 h, respectively, the fungal strains at 28 °C. After the incubation, the quantitative growth of the microbial colonies was evaluated, by determining the probable total number of bacterial colonies developed on the culture medium (UFC/mL). Bacterial colony counting was done with the Flash & Go automatic colony counter from Yul Instruments, Spain.

## 3. Results and Discussions

### 3.1. Synthesis and Characterization Materials

#### Scanning Electron Microscopy, SEM with Energy Dispersive X-ray Analysis, EDX

**Figure 1:**
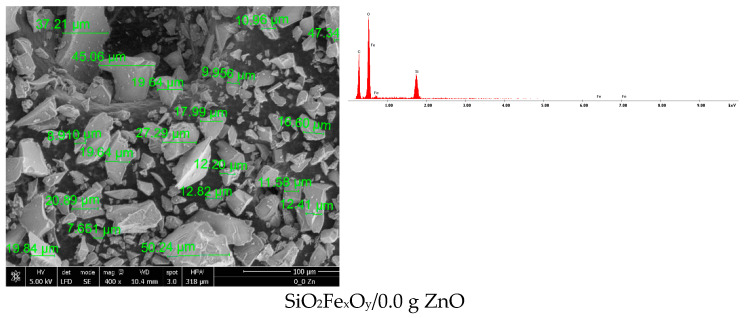

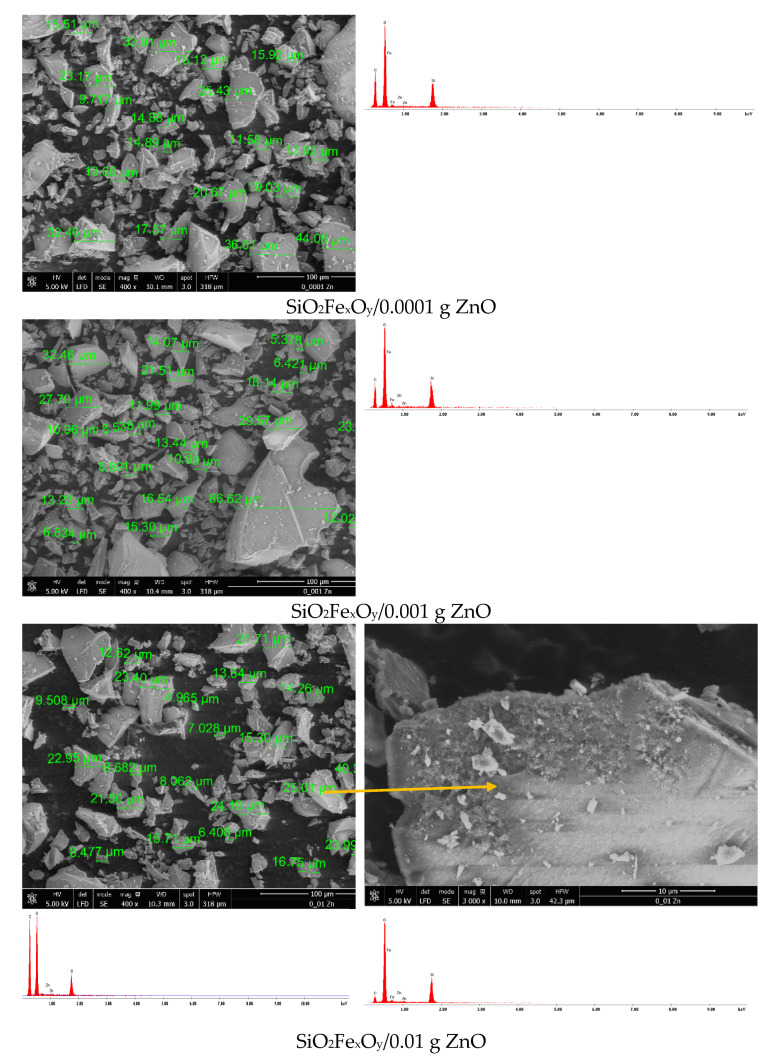

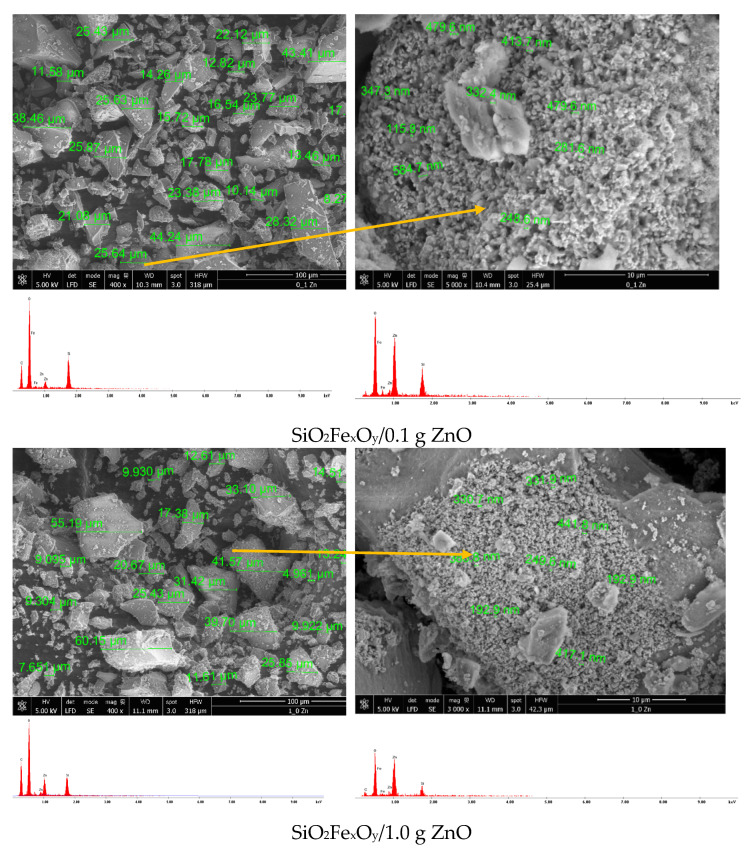
Scanning electron microscopy (SEM) and energy dispersive X-ray spectroscopy (EDX) for materials.

The morphology of synthetized materials can be observed in Figure 1, where scanning electron microscopy (SEM) and energy dispersive X-ray spectroscopy (EDX) are presented.

It can be seen from the SEM images that with the addition of a small amount of ZnO (0.0001 g ZnO and 0.001 g ZnO), the particles of the synthesized material do not significantly change their size or morphology. With the increase in the amount of ZnO added, starting with the material containing 0.01 g of ZnO, the surface of the material particles begins to undergo structural changes, first taking the appearance of a porous material. In the materials containing 0.1 g ZnO and 1.0 g ZnO, respectively, the surface of the particles is very obviously modified, observing the formation of clusters of nanometric dimensions, containing ZnO, on the surface.

### 3.2. Fourier-Transform Infrared Spectroscopy, FT-IR

**Figure 2:**
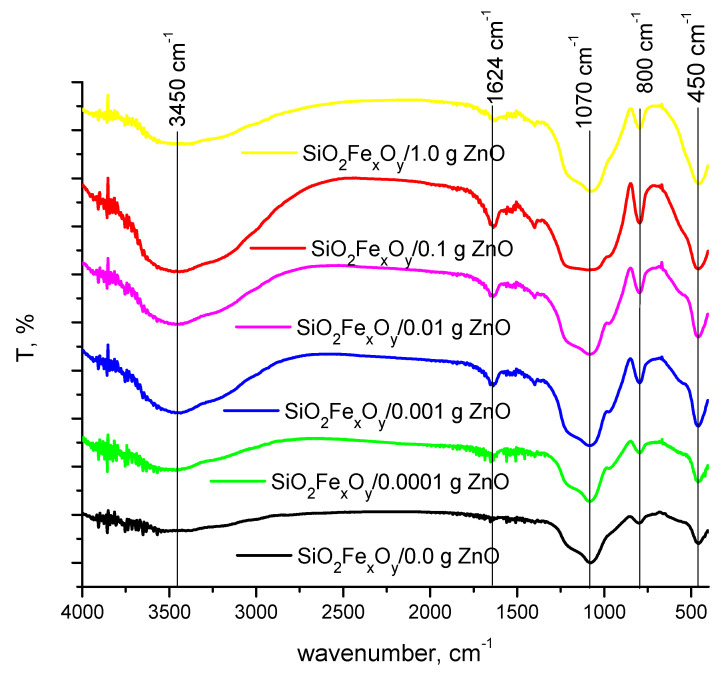
Fourier-transform infrared spectroscopy (FT-IR).

Figure 2 shows the FT-IR spectra of SiO_2_Fe_x_O_y_ materials with ZnO and without ZnO.

It can be seen from Figure 2 that the FT-IR transmittance spectrum (400 to 4000 cm^−1^) confirms the presence of Fe_x_O_y_SiO_2_ particles. Thus, the broad peaks located at 3400–3500 cm^−1^ and at 1600 cm^−1^ are specific to the presence of the O-H group [27,28,29,30]. Also, the wave numbers at 1000 cm^−1^, but also at 800 cm^−1^, can be attributed to the specific asymmetric and symmetric bond Si-O-Si [27,31].

At the wave number of 500 cm^−1^, the specific vibrations of the ZnO group overlap with those of Fe_x_O_y_SiO_2_, but it can be observed that, with the increase in the amount of ZnO, the peak is higher [32].

### 3.3. BET Specific Surface Area

Analyzing the data of textural parameters from N_2_ adsorption–desorption isotherms, we obtain the values of surface area (calculated using the BET (Brunauer–Emmett–Teller) method), average pore size (using the adsorption and desorption branch) and total pore volume (calculated from the last point near 1P/Po). The samples were degassed at 80 °C for 15 h in vacuum and analyzed using nitrogen at 77K.

In Table 1, the values calculated with computational methods for textural parameters are presented. 

Based on recorded adsorption isotherms (data not presented in the present paper) and according to IUPAC [33], the obtained materials present type IVa isotherms. After adding zinc oxide to the material, changes in its structure can be clearly observed, when the adsorption isotherms were changed from type H3 to type H2b. This type of hysteresis is specific for materials in which pore blocking occurs, having different dimensions and being much wider at the end.

It is noticed from Table 1 that the values for surface area are increasing with the decrease of the ZnO amount, reaching the highest values for sample 0.0001g ZnO. For this sample, it was shown that it presents pores smaller than 250 nm. 

In the case of all samples, it can be observed that the average pore size is around 6.5 nm. Important changes can be observed when the ZnO was introduced as: at low ZnO quantities, the surface area and the average pore size decrease slower, so the addition of ZnO has no major impact for the porogenity. With the increase of ZnO quantity upper than 0.01 g (in the case of materials with 0.1 g ZnO and 1.0 g ZnO), the ZnO has a porogen role; even if the surface area decreases, the average pore size increases.

The total pore volume indicates clearly that when the ZnO was added the values are starting to decrease, meaning that the pores are being filled.

XRD diffraction spectra

**Figure 3:**
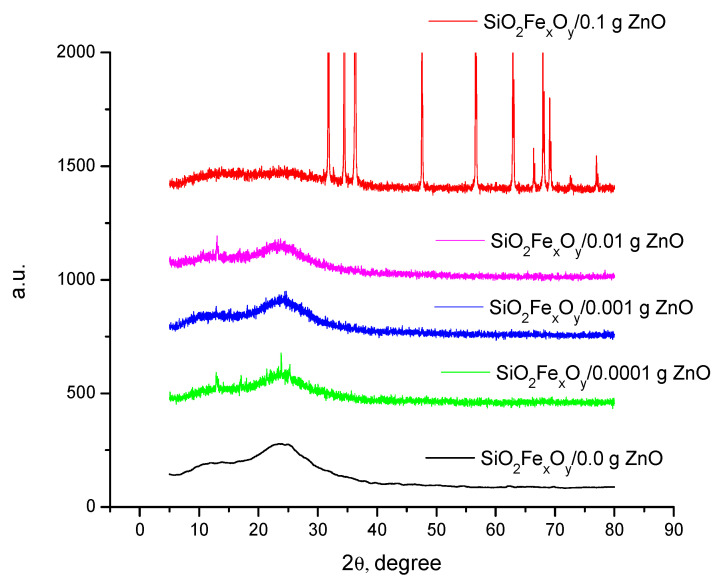
Recorded XRD spectra.

It is expected that, by changing the ZnO content, the material morphology changes. Recorded XRD spectra are depicted in Figure 3.

Analyzing the data presented in Figure 2, it can be observed that the major changes are taking place after 0.1 g of ZnO were added into the composite mass. Starting from the initial iron sample and comparing it to the data found in the database, it can be concluded that, through the used synthesis route, was obtained the maghemite cubic phase, concomitant with the iron acetylacetonate II orthorhombic phase. Besides these, the amorphous phase of silica is highlighted at 25 2θ [34]. After introducing and increasing the concentration of zinc oxide, it is observed that major changes occur only at the concentration of 0.1g ZnO in the material [35].

### 3.4. pH Point of Zero Charge, pH_pZc_

**Figure 4:**
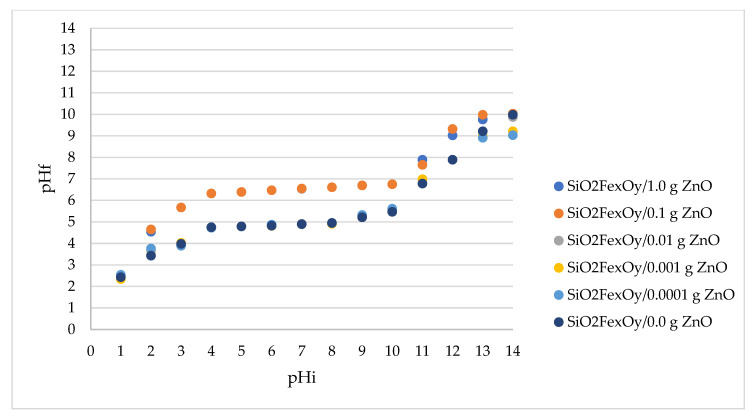
pH_pZc_ of materials.

Doping the Fe_x_O_y_SiO_2_ material with different amounts of ZnO can change the electrical charge of the material surface. Thus, the determination of pH_pZc_ is essential to establish the surface charge. That is why the determination of the point of zero electric charge was carried out by the method of bringing to balance [36,37]. In Figure 4, the pH_pZc_ values are presented for different materials in which the SiO_2_Fe_x_O_y_ material was doped with different amounts of ZnO.

From the experimental data presented in Figure 4, it can be seen that the electrical charge on the surface of the SiO_2_Fe_x_O_y_ material doped with small amounts of ZnO remains similar. For these materials, having ZnO in the range of 0.0001 g–0.01 g, the pH_pZc_ is ~4.9. For materials with 0.1 g ZnO and 1.0 g ZnO, the pH_pZc_ increases significantly, becoming ~6.3. It is known that if you work at a higher pH than pH_pZc_ values, the surface is negatively charged, allowing the adsorption of cationic species, and at a lower pH than pH_pZc_ values, the surface is positively charged, allowing the adsorption of anionic species.

### 3.5. Microbiological Tests

#### Antimicrobial Effect of Synthesized Materials on a Heterotrophic Bacterial Inoculum

The synthesized material is a xerogel formed from a matrix of mixed metal oxides (Fe_x_O_y_, SiO_2_), to which, to improve the antimicrobial properties, ZnO is added. In this regard, the material based on mixed metal oxides (Fe and Si) and then the material containing, in addition, ZnO were tested, under the aspect of antimicrobial potential.

In terms of the degree of toxicity of metal oxides, based on existent studies [38,39] and accordingly with the ecotoxicological database NanoE-Tox [40], ZnO has a higher toxic potential than iron oxides, implicitly, in terms of the antibacterial effect, it exhibits a superior antibacterial effect [41]. Of course, the antibacterial effect is dependent on the amount of metal oxide presented in the tested material.

**Figure 5:**
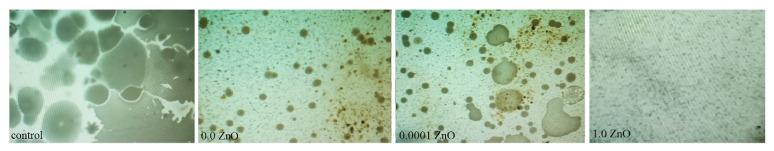
Morphological aspects of colonies growth.

In terms of morphological aspect (Figure 5), it is noted that in the plates with SiO_2_Fe_x_O_y_ without ZnO appear an inhibition of microbial growth, the colonies developed being numerous, but punctuated. Such behaviour is attributed to the antimicrobial effect of Fe and Si metal oxides. We can talk about a bacteriostatic effect, since the material failed to kill the bacteria but inhibited their growth, compared to the control sample.

With the addition of zinc oxide, at small amounts of ZnO (0.0001 g ZnO, 0.001 g ZnO, 0.01 g of ZnO), some colonies show better growth compared to those on plates without ZnO, but much lower growth compared to the control sample. This consideration suggests that, in small quantities, the antimicrobial effect of ZnO is “masked” by the stimulatory effect of Zn^2+^ ions on the growth of bacteria in the heterotrophic inoculum. Moreover, the data are supported by the fact that Zn^2+^ ions have a catalytic and structural role in cells [42].

**Figure 6:**
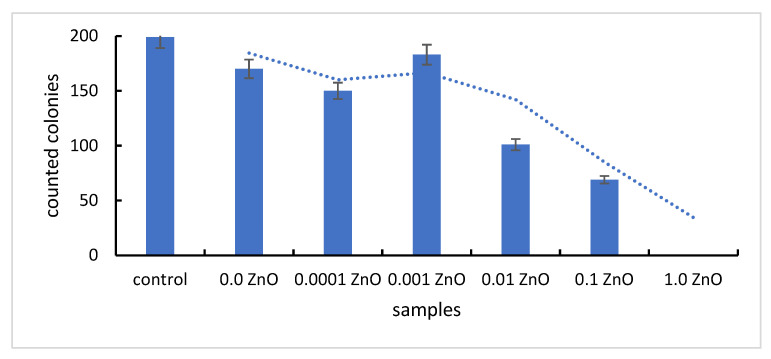
The colonies number evolution on the plates with various ZnO contents.

With an increase of the ZnO amount above a “threshold”, equivalent to the amount of ZnO tolerated by the bacterial cells, the manifestation of the destructive effect of ZnO is observed, materialized by the substantial reduction of the number of bacterial colonies identified on the Petri plates. With the addition of 1.0 g ZnO, the inhibition of bacterial growth is total, proven by obtaining Petri plates without any bacterial colony (Figure 6).

### 3.6. Antimicrobial Effect of Synthetized Materials on References Strains

**Figure 7:**
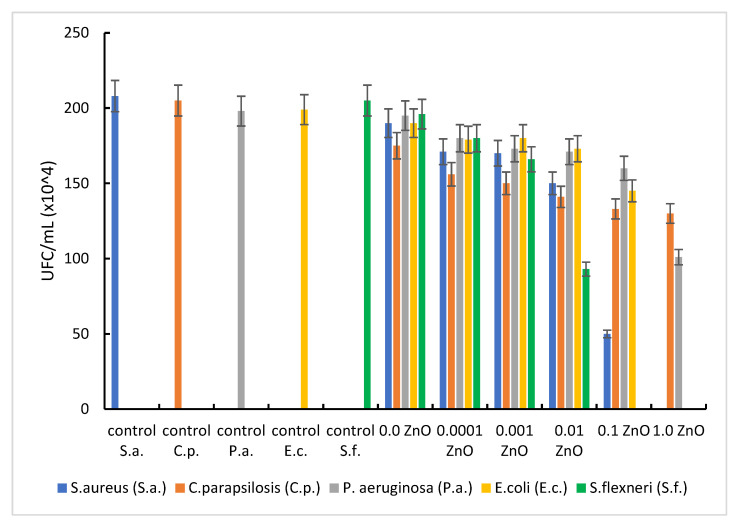
Comparative studies of ZnO on ATCC cultures.

The properties of composites consisting of oxide particles dispersed in an inert matrix depend significantly on the size and distribution of the particles, but also on the morphology and porosity of the matrix. Generally, samples containing ZnO showed a more pronounced antimicrobial activity against Gram-positive microorganisms (*S. aureus*) than against Gram-negative ones (*E. coli*, *S. flexneri*, and *P. aeruginosa*) (Figure 7). The result can be explained by the differences between the cell wall structure of Gram-positive and Gram-negative bacteria that interact with ZnO, accordingly with the point of zero charge (pH_pZc_) of the tested materials.

The pH_pZc_ for the material without ZnO (SiO_2_Fe_x_O_y_) is approximately 4.9. With the addition of ZnO, at very small amounts of ZnO, the pH_pZc_ does not change. In materials containing 0.1 g ZnO and 1.0 g ZnO, pH_pZc_ increases substantially, reaching 6.3. As mentioned above, at pH > pH_pZc_, the surface of the material is negatively charged, repelling anionic species and retaining predominantly cationic species. At pH < pHpZc, the surface of the material is positively charged, repelling cationic species and retaining predominantly anionic species. Consequently, working at pH 7.2, the charge of the ZnO-free material is negative, implying a weak interaction of the tested material with anionic species and a good interaction with cationic species in the bacterial membrane, ZnO interacting predominantly with cationic membrane constituents. In the case of materials with pH_pZc_ = 6.3, a value closer to the working pH, the electrical charge of the materials tends to be zero, when neutral species are mainly adsorbed, but adsorption of both cationic and anionic species is not excluded.

Iron being a major component in the production and metabolism of free radicals in biological systems, at physiological pH, Fe(II) is soluble, while Fe(III) precipitates. On the other hand, Fe(II) is unstable in aqueous media and tends to react under aerobic conditions with molecular oxygen to form Fe(III) and superoxide [42], subsequently involved in the production of H_2_O_2_, in ROS metabolism. The generation of anionic radicals, induced by the presence of metal radicals, has the effect of attacking DNA and other cellular components highly sensitive to oxidation, such as unsaturated fatty acids or phospholipid residues on the surface of the cell membrane.

The cell wall of Gram-positive bacteria is represented by a thick layer of peptidoglycan, in which teichoic acids, lipids and surface proteins are fixed, all of them acting as a barrier and protect the cell. Under these conditions, the antibacterial effect observed on this type of bacteria can be attributed to the oxidative stress generated by the production of ROS; ROS, including superoxide radicals (O^2−^), hydroxyl radicals (–OH), hydrogen peroxide (H_2_O_2_), and singlet oxygen (^1^O_2_), which can cause damage to bacterial proteins and DNA.

In the present study, the metal oxides involved in the synthesis, especially ZnO, could be the source that generated ROS that led to the inhibition of most pathogenic bacteria. A similar process has been described in other research where Fe^2+^ or Zn^2+^ reacted with oxygen to create hydrogen peroxide (H_2_O_2_) [25,43,44]. H_2_O_2_ is able to penetrate the bacterial membrane [42,45] and, under the actions of peroxidases, it can cause the destruction of cellular components, proteins, lipids and bacterial DNA, respectively.

The formed anionic radicals (HO^−^, respectively O^2−^) are negatively charged, which cannot infiltrate the cytomembrane of Gram-positive bacteria and remain on the surface of the bacterial cell, having the effect of attacking cellular components extremely sensitive to oxidation, such as unsaturated fatty acids or phospholipid residues on the surface of the cell membrane causing its destruction over time.

In Gram-negative bacteria, such as *E. coli*, *P. aeruginosa* and *S. flexneri*, the cell wall still has an outer membrane containing lipopolysaccharides, purines and a thin layer of peptidoglycan. Therefore, the better activity of ZnO-doped materials in the case of Gram-positive bacteria compared to those of Gram-negative bacteria can be attributed to the existence of this additional outer membrane in the case of Gram-negative bacteria, which makes it difficult to attack ROS generated by oxide metals involved in synthesis. The higher toxicity of ZnO when used in a higher amount for doping Fe_x_O_y_SiO_2_ may occur due to the generation of higher concentrations of ROS, which are released by ZnO on the surface of bacterial cells.

Another possible explanation for the better antibacterial activity against Gram-positive bacteria is that the ZnO-doped composite materials have a high molecular weight and cannot easily penetrate through the porins in the outer membrane, which makes the antibacterial effect delayed, or a larger amount of ZnO would be required to act on the Gram-negative bacterial cell. In other words, overcoming antibiotic resistance in Gram-negative bacteria is a more difficult task.

**Figure 8:**
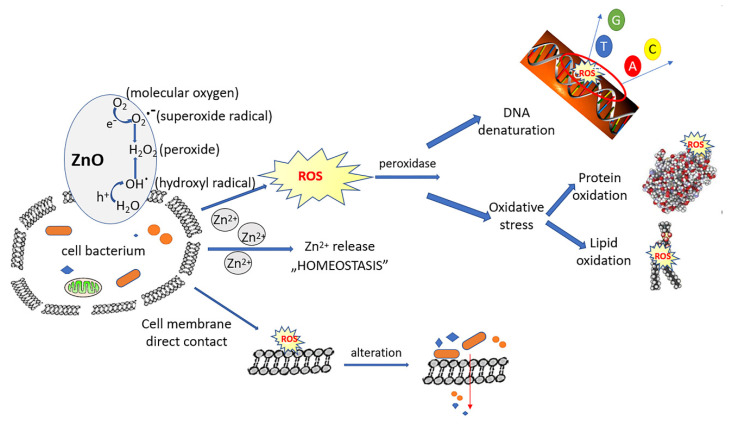
Proposed mechanism for antimicrobial effect.

Some bacteria have formed mechanisms to regulate the uptake and efflux of metal ions at the cellular level to maintain stable intracellular ion concentration. On the other hand, once a certain amount of ZnO is exceeded, the Zn^2+^ ions themselves may be responsible for antimicrobial activity [46], as the hydrolysis of ZnO into Zn^2+^ ions has been reported in several studies [4,46]. Although the Zn^2+^ ion has a catalytic and structural role, homeostasis of this ion is important [47], as harmful effects appear in excess. This is how the behaviour of *S. flexneri* can be explained under the action of the material doped with 0.001 g ZnO and 0.01 g ZnO when there is still good bacterial growth, respectively, in the case of the material containing 0.1 g ZnO when there is no growth of the bacterial colony. By correlating obtained data with the information found in the scientific literature was proposed a mechanism which explains the antimicrobial effect of new prepared material (Figure 8).

**Figure 9:**
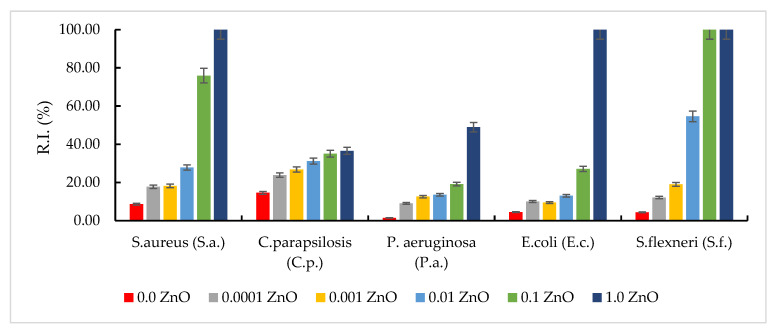
Microbial inhibition rate at various ZnO quantities.

In the same way, *E. coli* can tolerate a larger amount of ZnO (up to 0.1 g), total antibacterial effect being observed in the material with 1.0 g ZnO. If in 0.0001 g ZnO material the inhibition rate is about 10%, with the increase in the concentration to 0.1 g ZnO (10^3^ times) an inhibition rate of microbial growth of about 27% is manifested, and with an increase of another 10 times the amount of ZnO (1.0 g ZnO), a total antibacterial effect occurs (Figure 9).

In the case of *P. aeruginosa*, a weaker antibacterial effect was observed, regardless of the amount of ZnO. However, the good bactericidal effect obtained by comparing the sets of experiments with plates containing 0.1 g ZnO and 1.0 g ZnO can be emphasized. If by increasing the amount of ZnO by 10^3^ times (from 0.0001 g ZnO to 0.1 g ZnO) the bacterial inhibition rate varied between 9 and 19%, when increasing the amount of ZnO by another 10 times (from 0.1 g ZnO to 1.0 g ZnO), 49% inhibition of bacterial growth was obtained. In this case, the extremely important role of the homeostasis of Zn^2+^ ions is found.

Regarding the low antifungal effect of the synthesized materials, proven by the growth, in a ‘steady state” of the tested fungal strain (*C. parapsilosis*), it is known that ROS activity in fungal cells is low, due to the low sensitivity of fungi to the action of generated free radicals.

## 4. Conclusions

Currently, there is a growing interest in nanoparticles of metals and metal oxides that can be used as agents with antibacterial potential. This is because the basis of the antimicrobial activity of metals lies in the ability of metal ions to inhibit enzymes, facilitate the generation of reactive oxygen species, cause damage to cell membranes, or prevent the absorption of microelements of vital importance by microbes.

To combat infections, a series of inorganic antimicrobial agents have been used over time, among which the use of metal oxides stands out. Although zinc is considered non-toxic to the body, as long as it is essential for the functioning of metalloproteins, increasing evidence suggests that free zinc ions can cause negative effects on cells. To eliminate the cytotoxic effect, zinc cations are linked with bioactive ligands (proteins) and ZnO is reached. In most studies conducted to demonstrate the effectiveness of ZnO in terms of antimicrobial potential, it has been emphasized that ZnO is non-toxic to human organisms.

The antibacterial effect due to the destructive role of ROS generated by metal oxides is crucial in obtaining the antimicrobial effect. Although the contact surface, in the case of samples with low ZnO content (600 m^2^/g), is much larger compared to that of samples with high ZnO content (306 m^2^/g), a very good antibacterial effect was obtained when the amount of ZnO was able to generate substantial amounts of ROS.

For *S. aureus*, the total antibacterial effect was obtained if the amount of ZnO added is 1.0 g. For Gram-negative bacteria, total antibacterial effect was observed on *S. flexneri* (for an amount of 0.1 g ZnO added), followed by *E. coli* (for an amount of 1.0 g ZnO added). For *P. aeruginosa*, the maximum inhibition rate obtained for the material with 1.0 g ZnO was approximately 49%.

Given the known resistance of these bacterial species to the action of various bactericidal compounds, we can consider that the synthesized material that showed a very good antibacterial effect (the one with 1.0 g of ZnO content) can represent a starting point for obtaining compounds with remarkable antibacterial potential, but for this requires adjusting/improving the composition of the constituents involved in the synthesis.

## Figures and Tables

**Table 1:** Textural parameters from N_2_ adsorption–desorption isotherms.

Name	Surface Area, BET, m^2^/g	Average Pore Size, nm	Total Pore Volume, cm^3^/g
SiO_2_Fe_x_O_y_/ 0.0 g ZnO	305	20.29	1.545 × 10^0^ cc/g for pores smaller than 139.8 nm
SiO_2_Fe_x_O_y_/ 0.0001 g ZnO	600	6.12	9.197 × 10^−1^ cc/g for pores smaller than 250.2 nm
SiO_2_Fe_x_O_y_/ 0.001 g ZnO	573	5.93	8.512 × 10^−1^ cc/g for pores smaller than 158.4 nm
SiO_2_Fe_x_O_y_/ 0.01 g ZnO	559	5.60	7.849 × 10^−1^ cc/g for pores smaller than 148.6 nm
SiO_2_Fe_x_O_y_/ 0.1 g ZnO	476	6.29	7.497 × 10^−1^ cc/g for pores smaller than 147.7 nm
SiO_2_Fe_x_O_y_/ 1.0 g ZnO	306	7.86	6.023 × 10^−1^ cc/g for pores smaller than 162.6 nm
